# Supplementary material for: Hallucinating structure-conditioned antibody libraries for target-specific binders
Source: Front Immunol. 2022 Oct 21;13:999034. doi: 10.3389/fimmu.2022.999034 (PMC9635398; doi:10.3389/fimmu.2022.999034)
Supplement: Supplementary file 1 [file DataSheet_1.pdf]

Supplementary Information: Hallucinating structure-conditioned antibody libraries for target-specific binders

Sai Pooja Mahajan[1], Jeffrey A. Ruffolo[2], Rahel Frick[1], Jeffrey J. Gray[1,2,3*]

[1]Department of Chemical and Biomolecular Engineering, Johns Hopkins University, Baltimore, Maryland 21218, United States

[2]Program in Molecular Biophysics, Johns Hopkins University, Baltimore, Maryland 21218, United States

[3]Sidney Kimmel Comprehensive Cancer Center, Johns Hopkins School of Medicine, Baltimore, Maryland, United States

*Corresponding Author

Supplementary Tables and Figures

SI Table 1. PDB ids of 20 antibodies selected from DeepAb Test set[1] for sequence recovery.

PDB IDs
1bey, 1cz8, 1dlf, 1fns, 1gig, 1jfq, 1jpt, 1mfa, 1mim, 1mlb, 1mqk, 1nlb, 1oaq, 1seq, 1sy6, 1yy8, 2d7t, 2e27, 2fb4, 2fbj

SI Table 2. Spearman correlation between perplexities of PyIgClassify cluster profiles and hallucinated profiles for CDR loops with and without wildtype seeding.

CDR	Wildtype Seeding		No Seeding	
	Spearman	p-value	Spearman	p-value
H1	0.55	< 0.001	0.439	< 0.001
H2	0.42	< 0.001	0.296	< 0.001
H3	0.25	< 0.001	0.261	< 0.001
L1	0.438	< 0.001	0.195	< 0.001
L2	0.08	0.096	-0.0	0.32
L3	0.437	< 0.001	0.363	< 0.001

SI Table 3. Percentage of hallucinated sequences that fold into the target structure for different modes of hallucination for the trastuzumab antibody CDR H3 loop.

Run	% Designs with RMSD ≤ 2 Å	
	DeepAb	IgFold
Unrestricted	54.5	43.6
Motif-Restricted (95 and 100A)	72.0	61.1
Motif-Restricted (95, 99, 100, 100A)	78.6	62.0
Motif- (95 and 100A) and Seq-Restricted	72.1	74.3

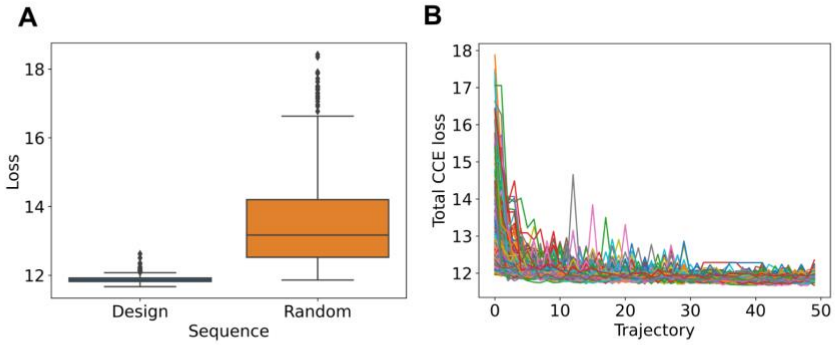

SI Figure 1. Hallucination minimizes geometric losses of designed sequences. A. Comparison of distribution of CCE loss of the designed sequences versus random sequences they were initialized with. B. Minimization of CCE losses over ~200 hallucination trajectories for an example CDR H3 loop.

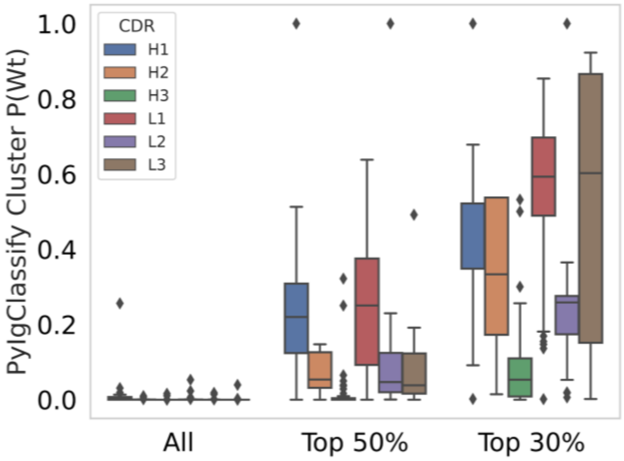

SI Figure 2. Likelihood of recovering wildtype sequence based on PyIgClassify sequence profile for cluster corresponding to wildtype sequence for "ALL" positions (or 100% sequence recovery) on the CDR, for "Top 50%" most conserved positions and for "Top 30%" most conserved positions.

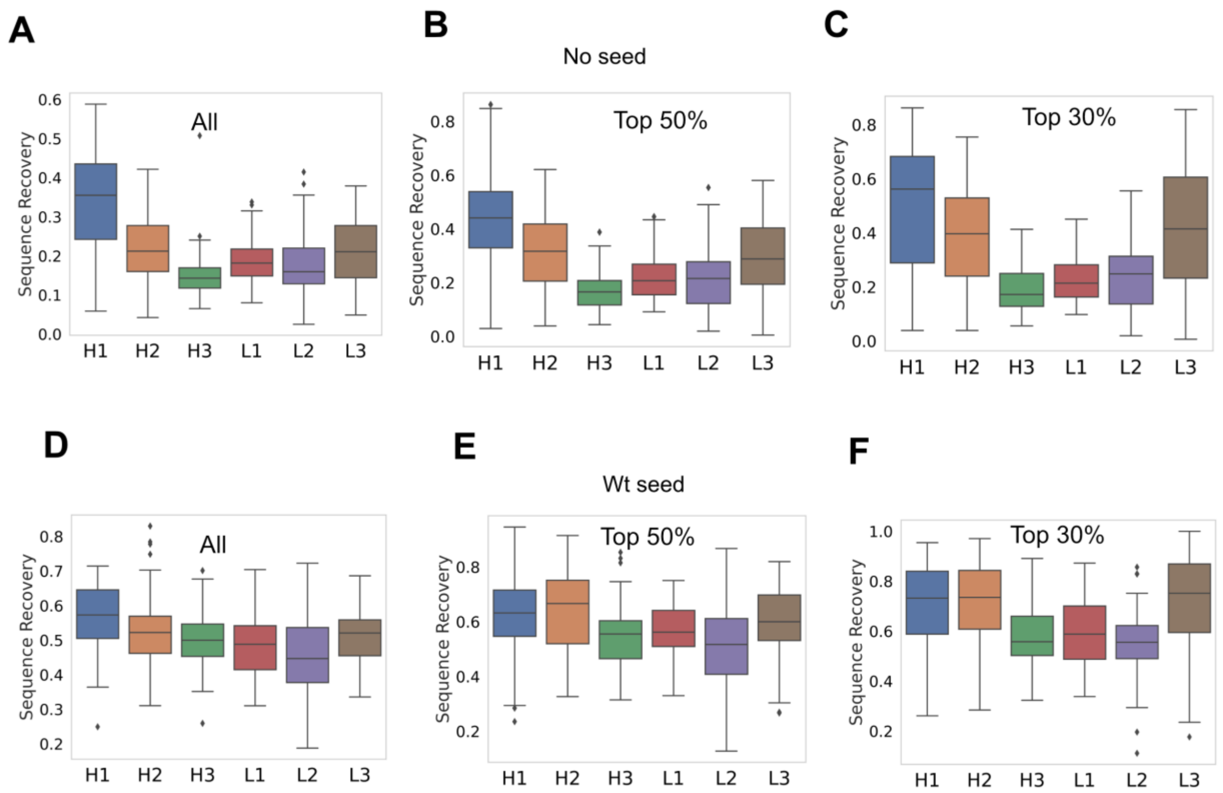

SI Figure 3. Sequence recovery for "All" positions, "Top 50%" most conserved positions and "Top 30%" most conserved positions (conservation estimated from PyIgClassify cluster profiles) for hallucination without seeding (A, B, C) and with seeding (D, E, F).

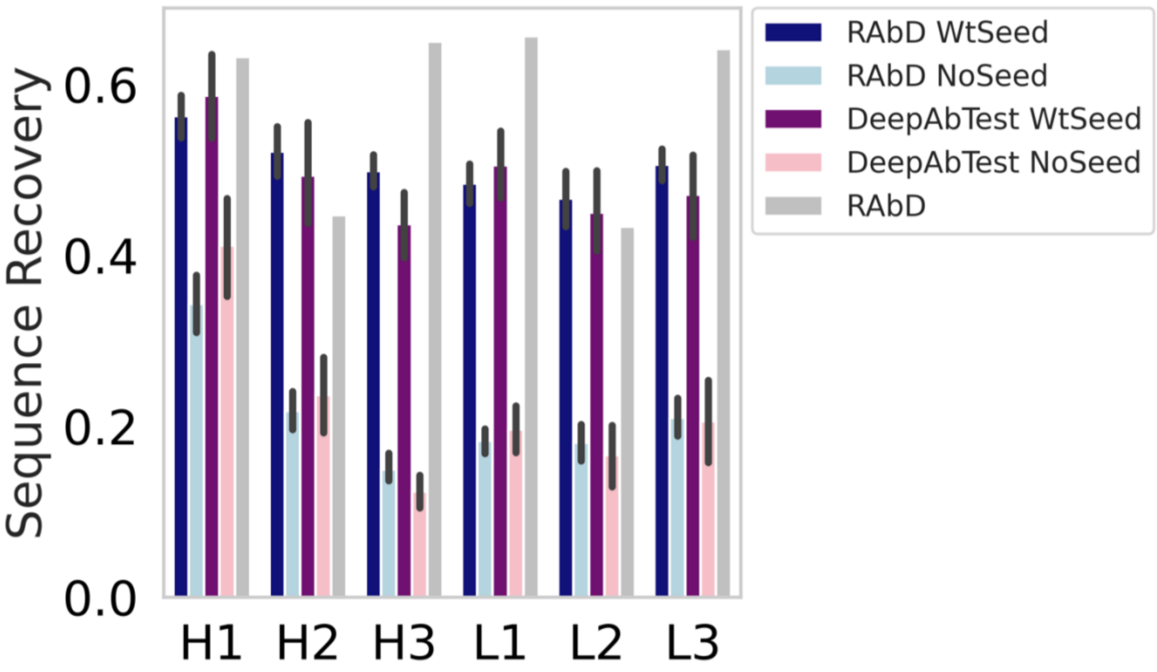

SI Figure 4. Sequence recovery with (DeepAbTest WtSeed) and without wildtype seeding (DeepAbTest NoSeed) on a benchmark set of 20 antibodies selected from the DeepAb test set. This benchmark set was "blind" to the pre-trained DeepAb model used in this work. The sequence recovery numbers are similar to those obtained for the RAbD dataset with and without wildtype seeding. The 54/60 antibodies in the RAbD dataset were also in DeepAb's training set. However, no difference is sequence recovery is observed between the DeepAb Test set and the RAbD dataset. We have also included the sequence recovery of the RAbD method on the RAbD dataset (RAbD) for comparison. See SI Table 1 for the pdb ids of 20 antibodies chosen from the DeepAb test set.

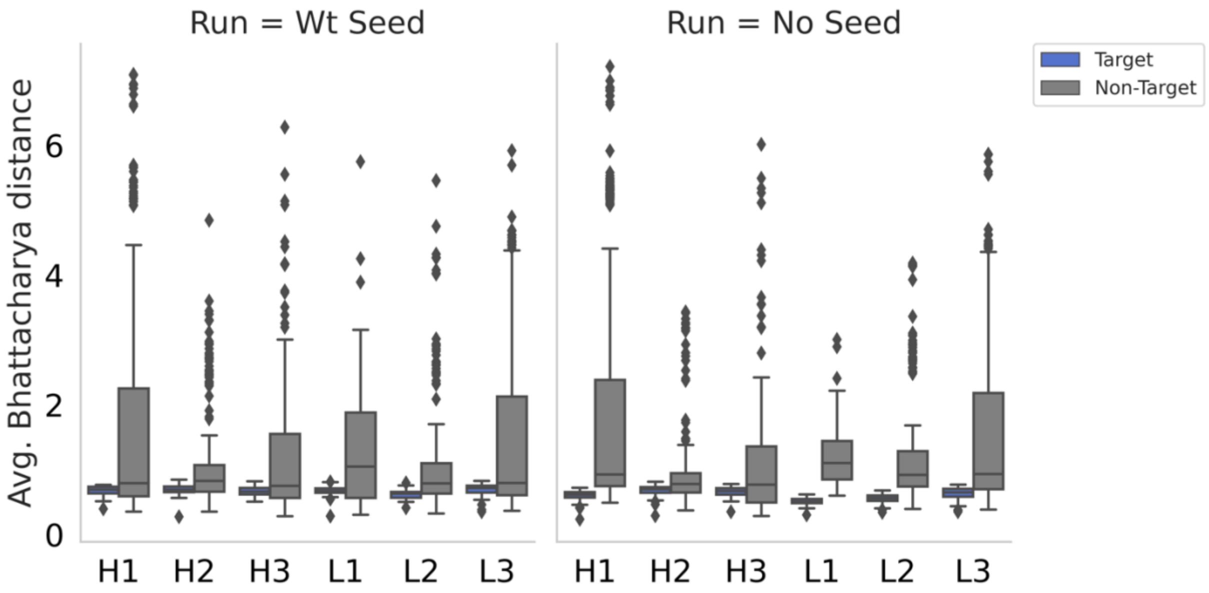

SI Figure 5. Average Bhattacharya distance (BD) between the hallucinated sequence and the Target and non-Target PyIgClassify clusters. BD is the negative logarithm of BC and a measure of the distance between two distributions. BD is first averaged over all hallucinated sequences for each target and then averaged over all targets for each CDR. Sequence profiles of hallucinated designs are closer to the target (blue) PyIgCluster than non-target (gray) clusters.

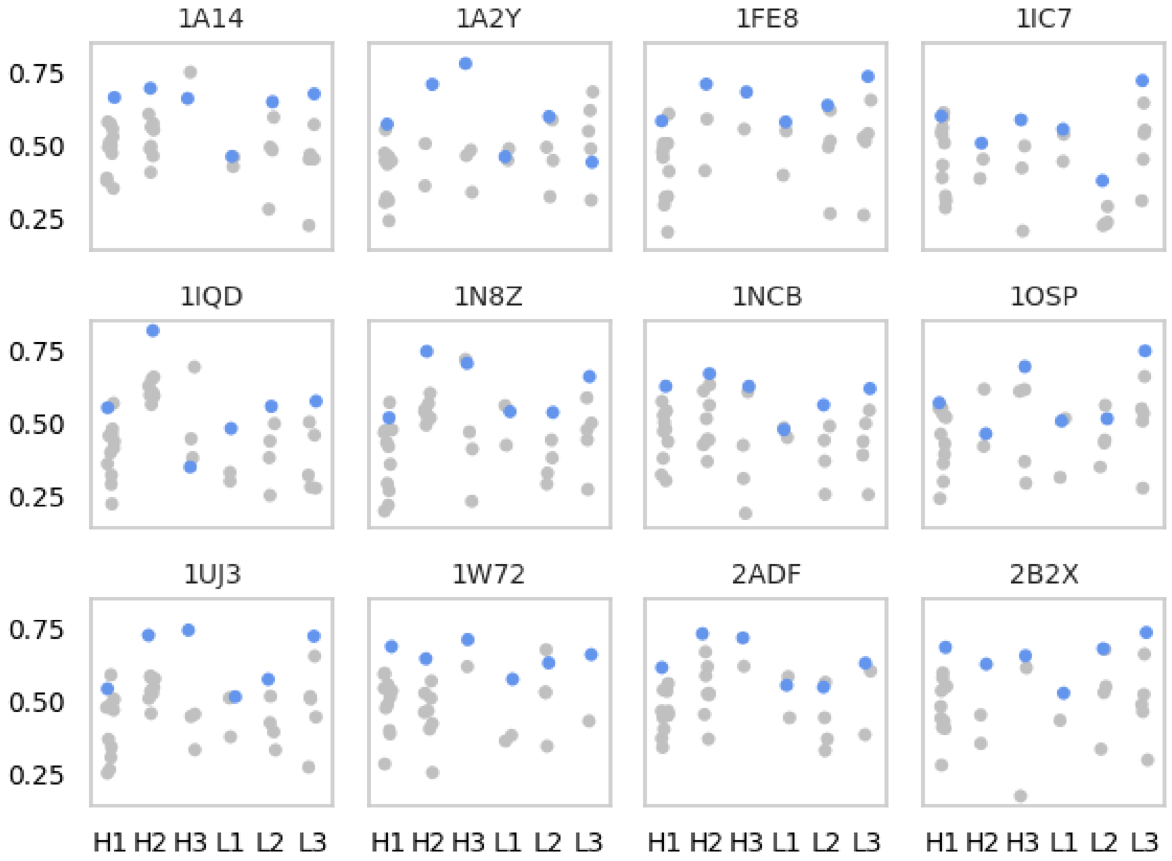

SI Figure 6. Bhattacharya coefficient averaged over all designed positions between the sequence profile from 50 hallucinated (no wildtype seeding) CDRs and the *target cluster (blue)* and non-target cluster (gray) for all 6 CDRs for targets 1-12 from the RAbD benchmark set.

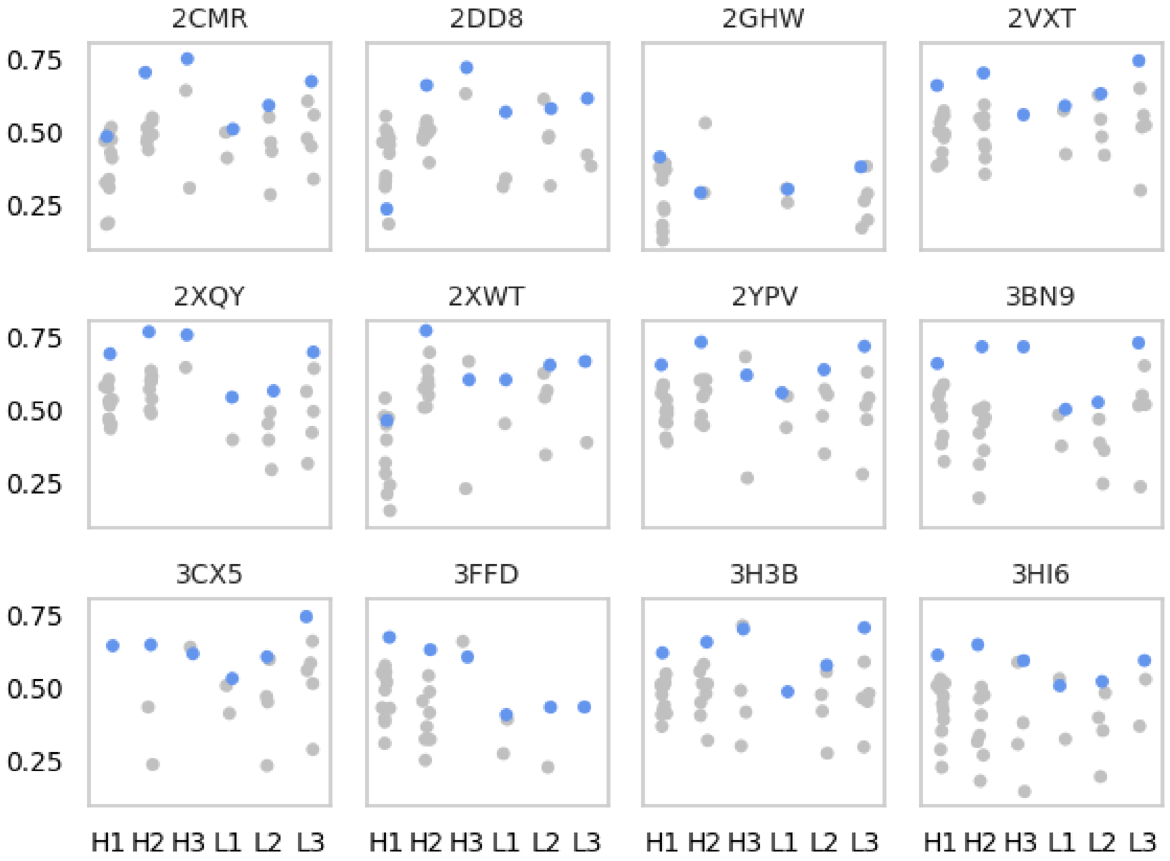

SI Figure 7. Bhattacharya coefficient averaged over all designed positions between the sequence profile from 50 hallucinated (no wildtype seeding) CDRs and the *target cluster (blue)* and non-target cluster (gray) for all 6 CDRs for targets 12-24 from the RAbD benchmark set. For 2GHW H3, the PyIgClassify target clusters are "starred" clusters. These clusters are considered unreliable and not considered in our analysis.

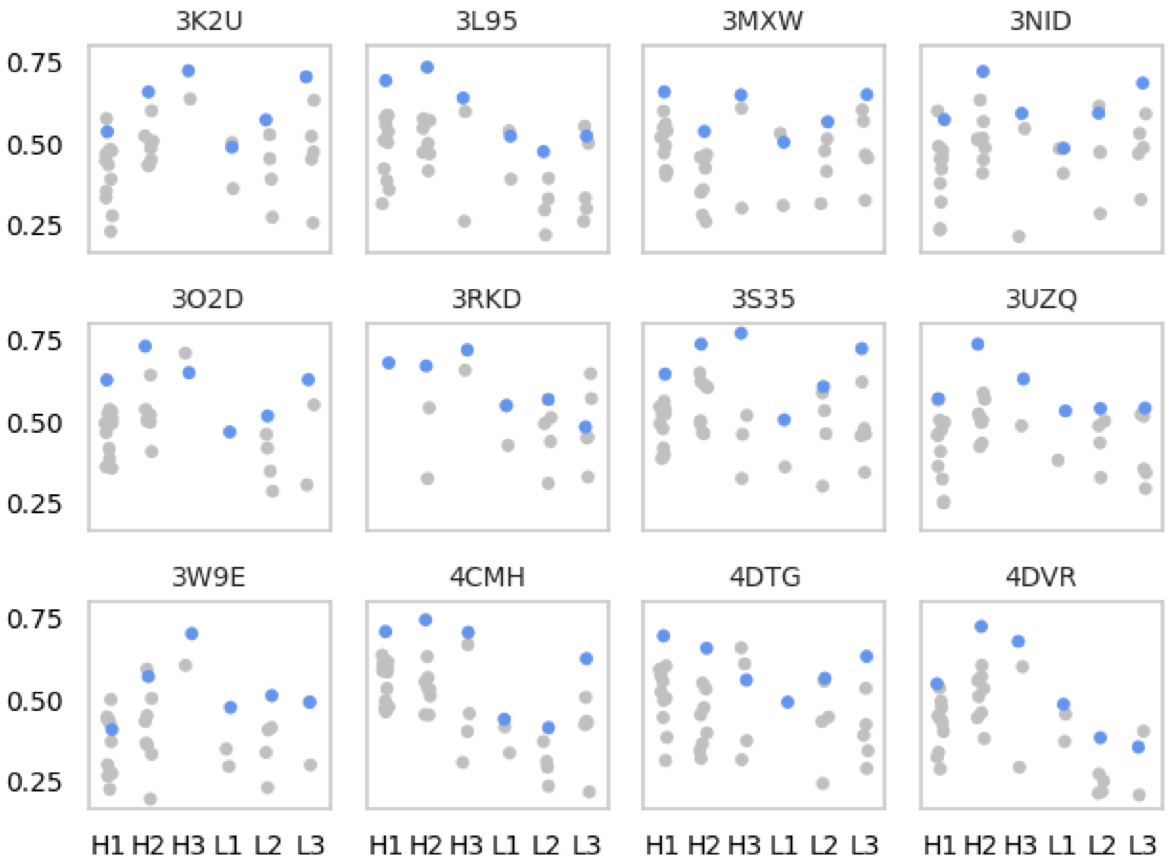

SI Figure 8. Bhattacharya coefficient averaged over all designed positions between the sequence profile from 50 hallucinated (no wildtype seeding) CDRs and the *target cluster (blue)* and non-target cluster (gray) for all 6 CDRs for targets 24-36 from the RAbD benchmark set.

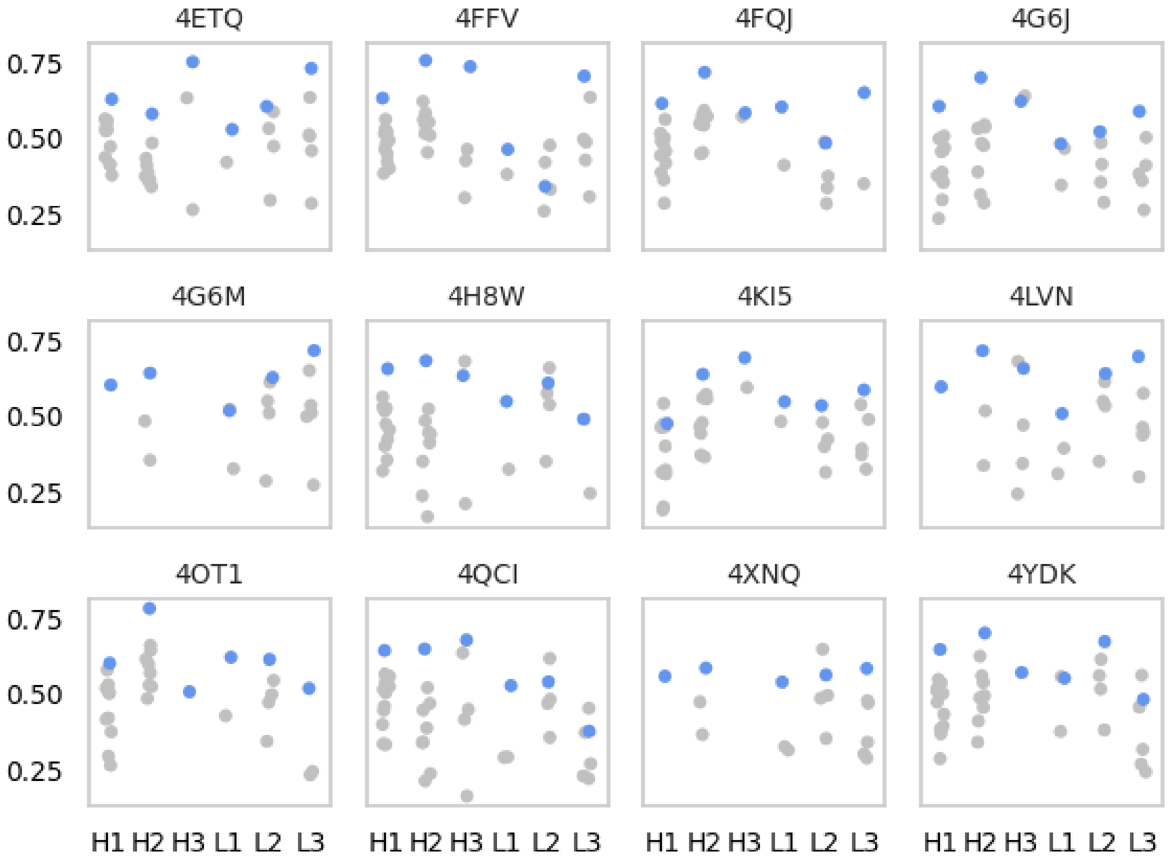

SI Figure 9. Bhattacharya coefficient averaged over all designed positions between the sequence profile from 50 hallucinated (no wildtype seeding) CDRs and the *target cluster (blue)* and non-target cluster (gray) for all 6 CDRs for targets 26-48 from the RAbD benchmark set. For 6G6M H3 and 4XNQ H3, the PyIgClassify target clusters are "starred" clusters. These clusters are considered unreliable and not considered in our analysis.

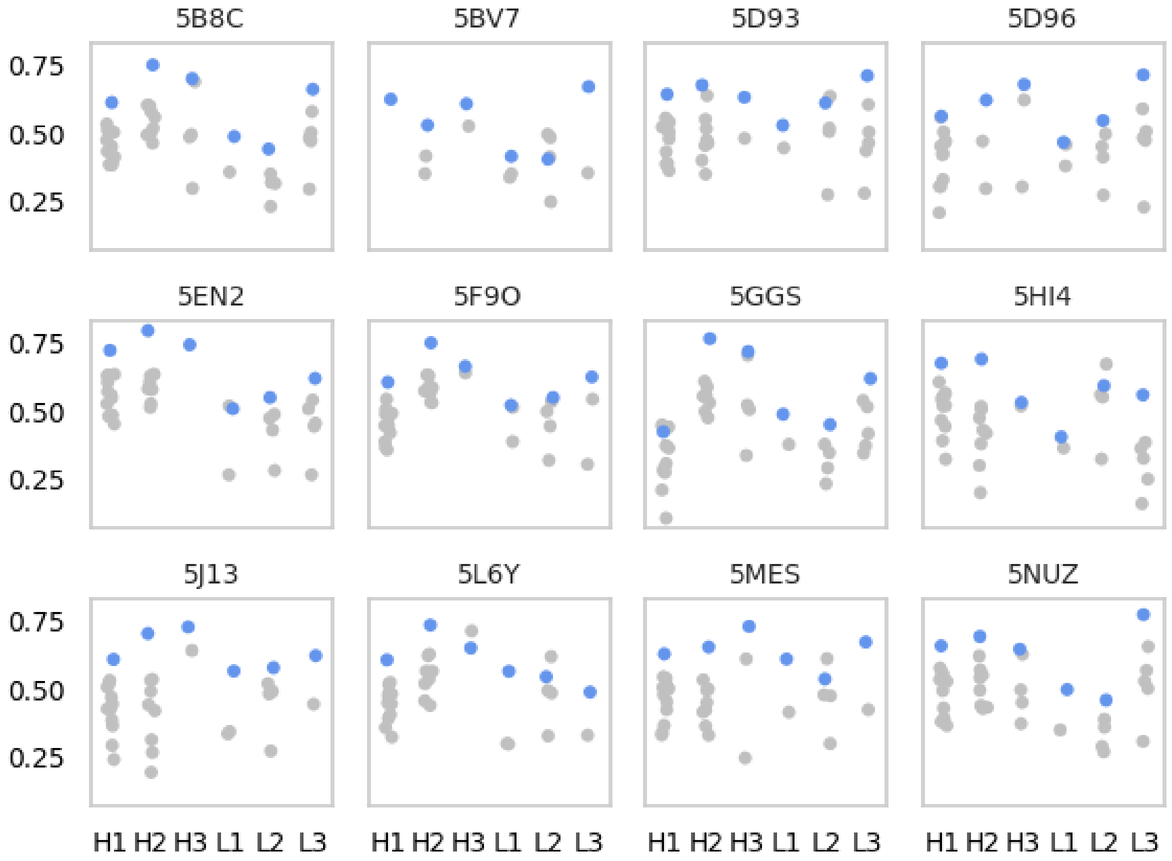

SI Figure 10. Bhattacharya coefficient averaged over all designed positions between the sequence profile from 50 hallucinated (no wildtype seeding) CDRs and the *target cluster (blue)* and non-target cluster (gray) for all 6 CDRs for targets 48-60 from the RAbD benchmark set.

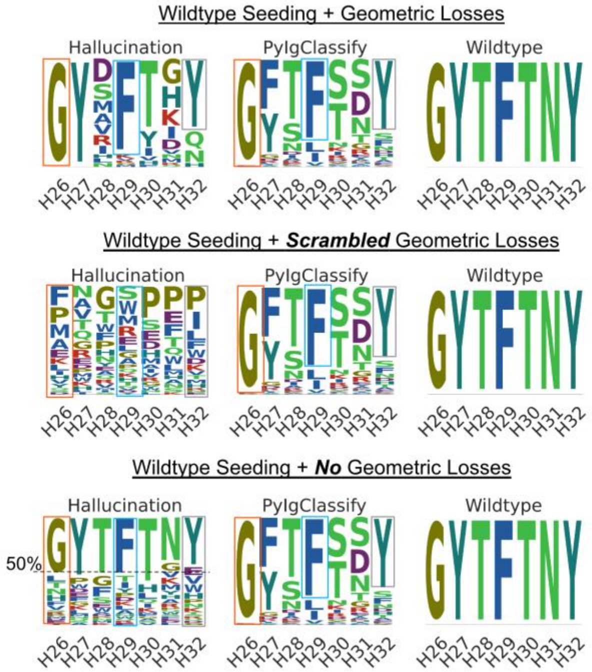

SI Figure 11. Effect of wildtype seeding and geometric losses on sampled profiles. Geometric losses guide hallucination to sample profiles resembling those of target structural clusters. When geometric losses are scrambled, wildtype seeding does not result in either retention of the wildtype sequence nor sampling of PyIgClassify target cluster-like profiles. Thus, wildtype seeding with incorrect geometric losses generates random sampling. When geometric losses are completely removed, the wildtype sequence is sampled about 50% of the times for all positions same as the degree to which the wildtype sequence is spiked in the initialization of the designed sequence. However, the sequence profiles do not resemble those observed for PyIgClassify clusters. For example, *with geometric losses (top panel)*, H26 almost always samples a glycine same as PyIgClassify. This is not true when geometric losses are absent.

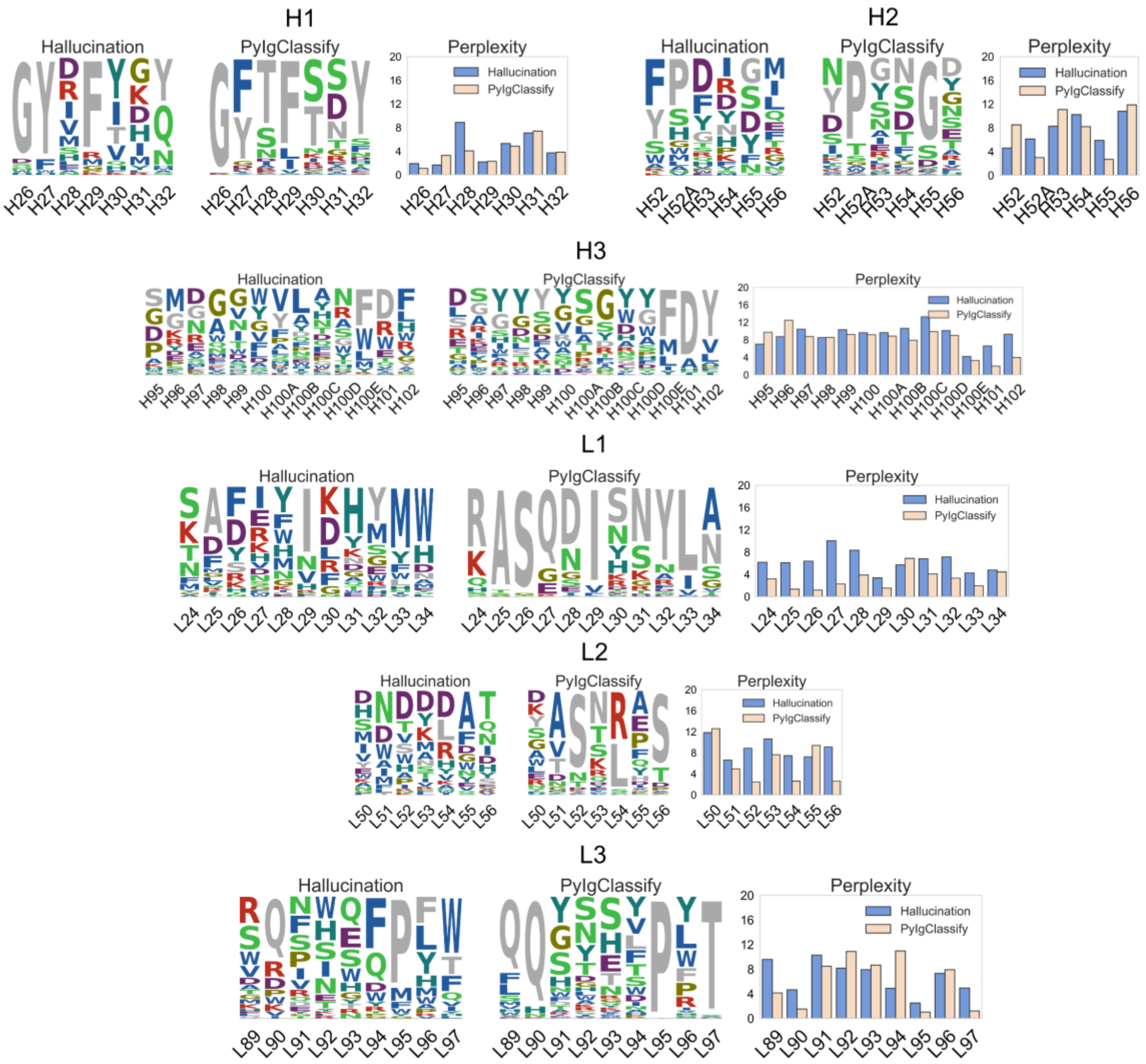

SI Figure 12. Comparison of perplexity of per-residue probability distribution derived from hallucinated designs (without wildtype seeding) for CDRs of antibody (PDB: 1A14, RAbD dataset) and the target PyIgClassify cluster. Wildtype sequence is shown in grey. Each sequence logo was constructed from 50 designs for CDRs H1, H2, L1, L2 and L3 and 100 designs for CDR H3.

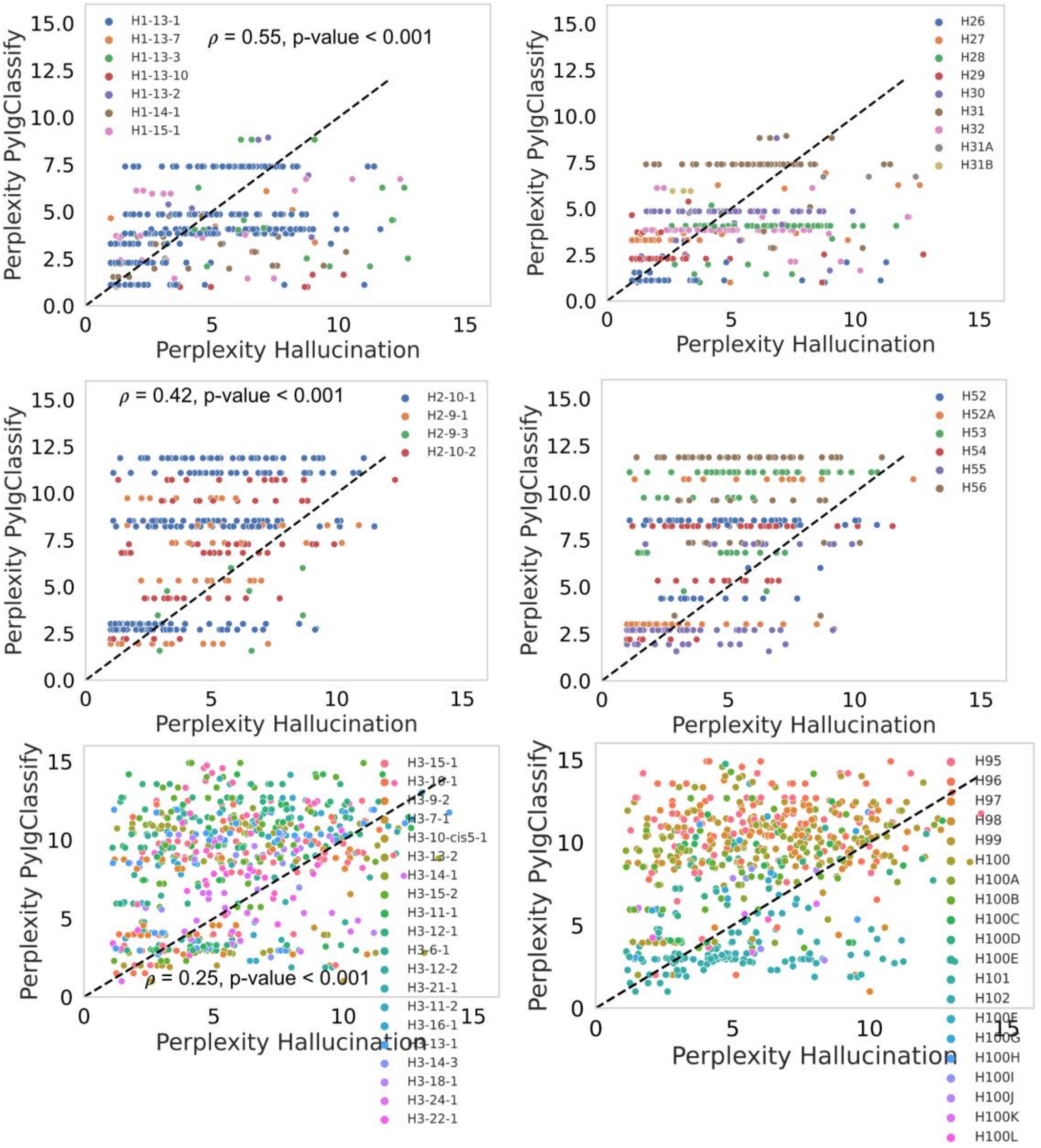

SI Figure 13. Comparison between perplexities of PyIgClassify cluster profiles and hallucinated profiles with wildtype seeding for CDRs H1, H2 and H3.

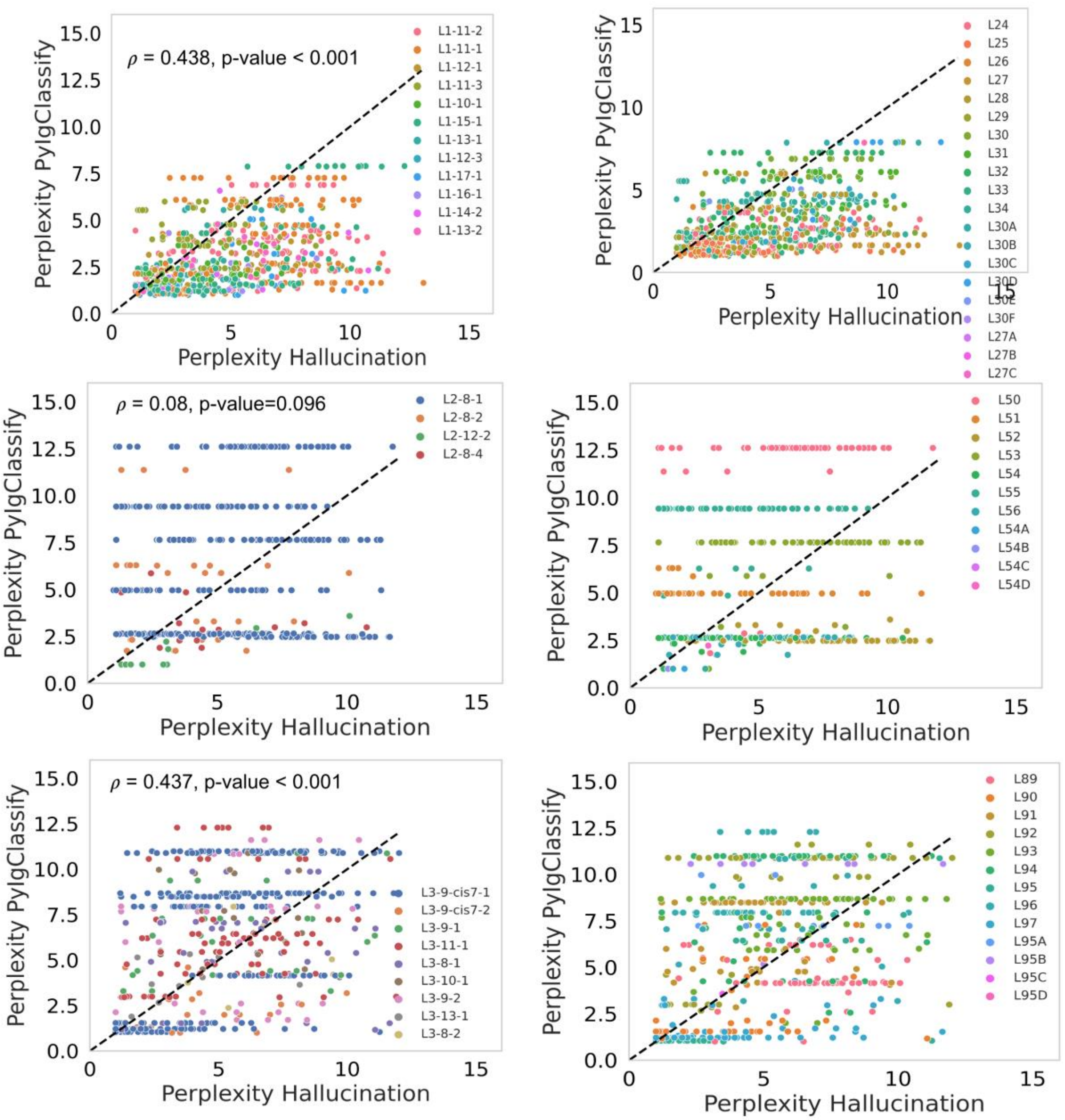

SI Figure 14. Comparison between perplexities of PyIgClassify cluster profiles and hallucinated profiles with wildtype seeding for CDRs L1, L2 and L3.

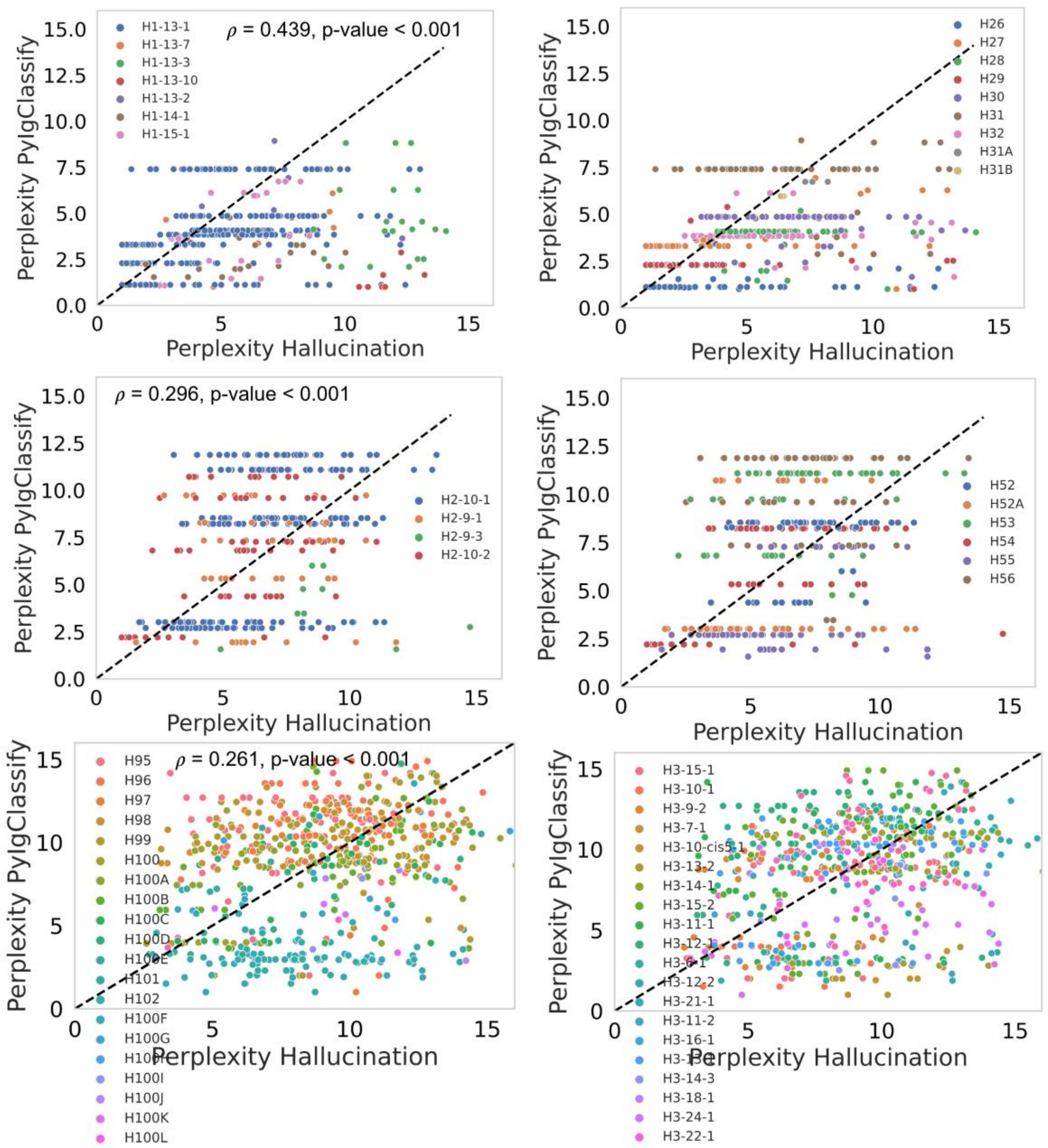

SI Figure 15. Comparison between perplexities of PyIgClassify cluster profiles and hallucinated profiles *without* wildtype seeding for CDRs H1, H2 and H3.

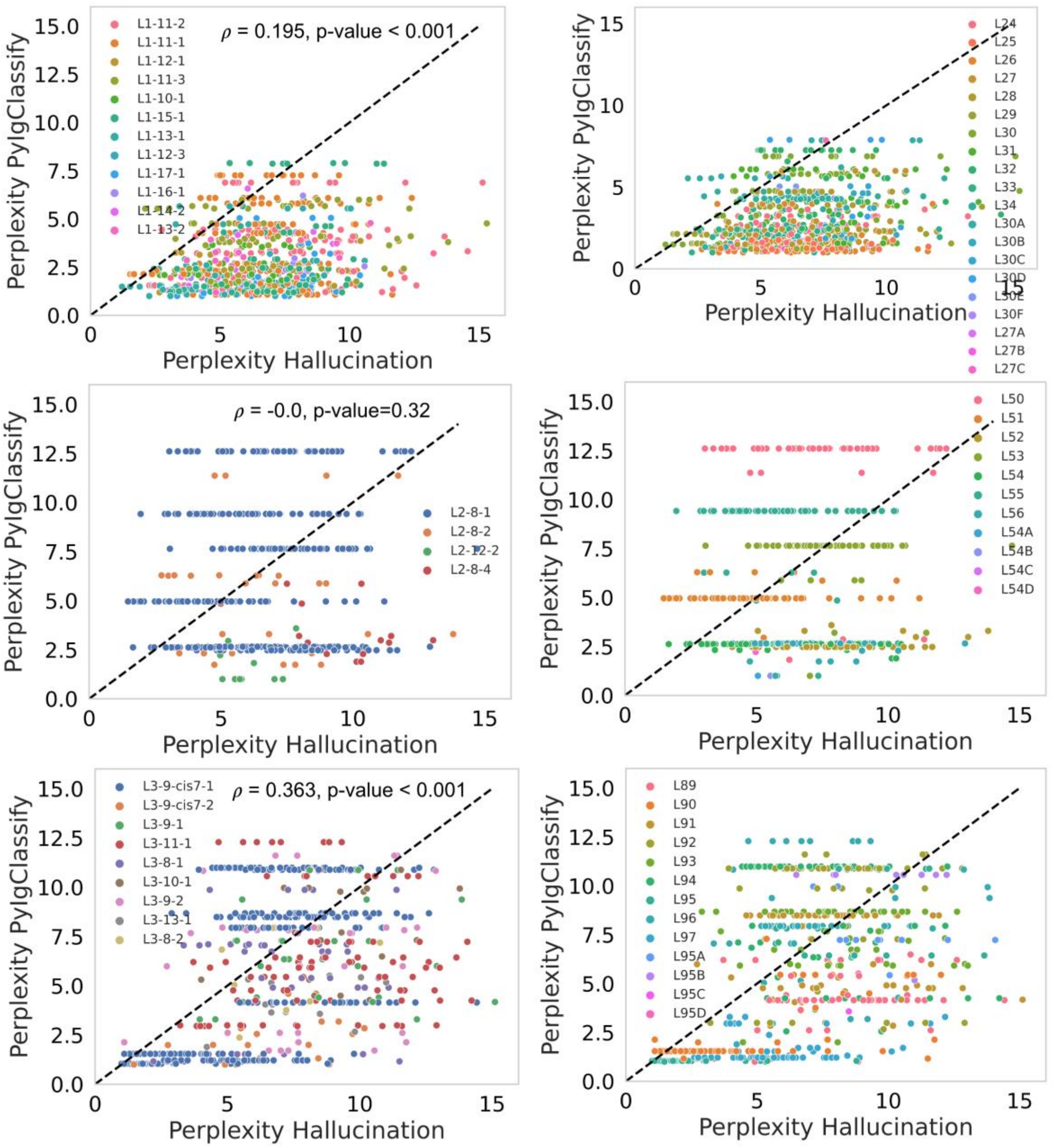

SI Figure 16. Comparison between perplexities of PyIgClassify cluster profiles and hallucinated profiles *without* wildtype seeding for CDRs L1, L2 and L3.

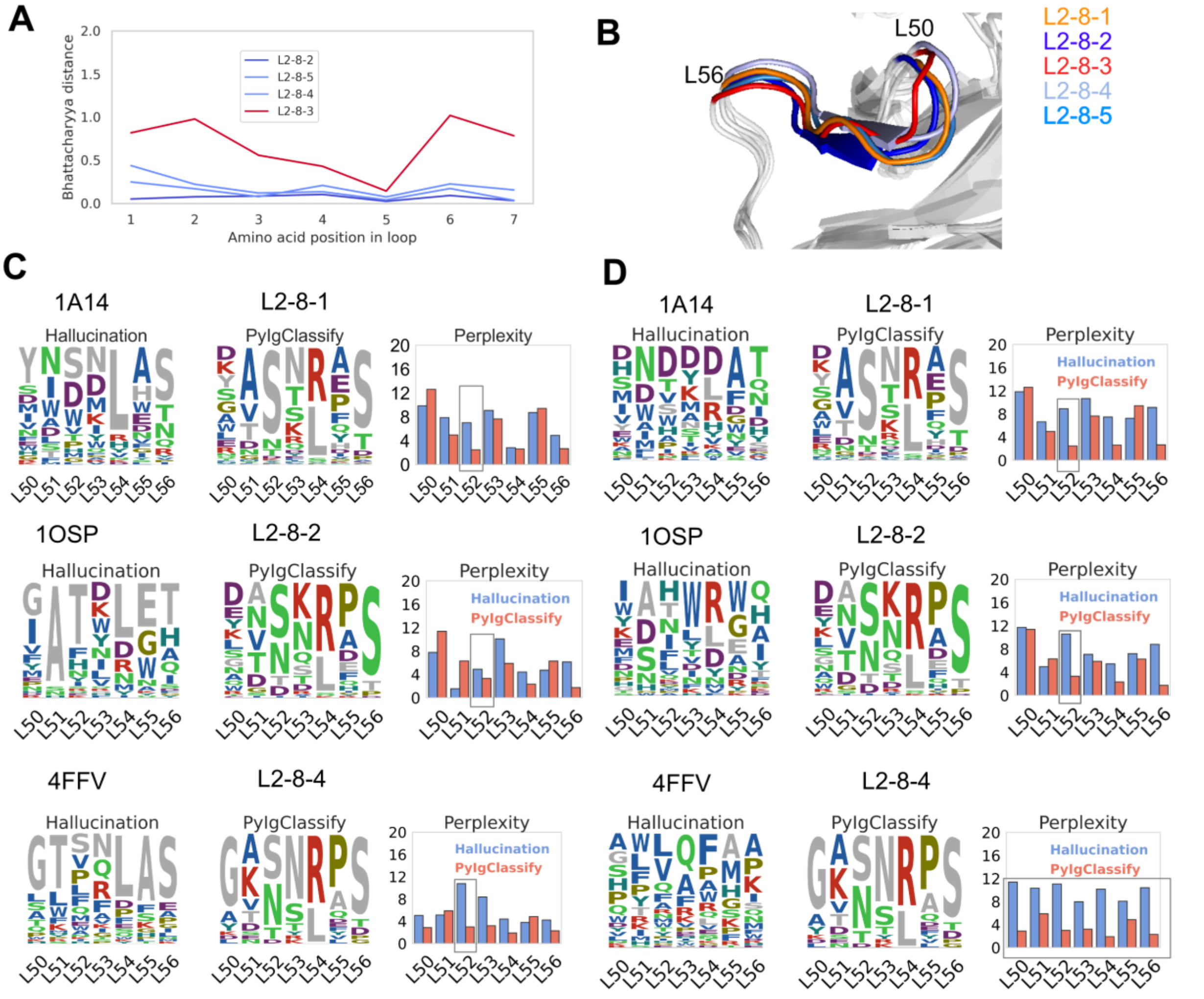

SI Figure 17. Comparison with CDR L2 loop clusters from PyIgClassify. A. Bhattacharyya distance of sequence profiles of PyIgClassify cluster L2-8-1 (the most represented dataset in the RAbD dataset-- 57/60 Abs belong to this cluster) from other L2 clusters of the same length. B. CDR L2 loop conformation of representative loops for each canonical cluster. Loop conformations for different clusters show significant overlap. C. and D. Sequences profiles and perplexity comparisons for three selected antibodies from the RAbD benchmark set. Hallucinated profiles (*C:* with wildtype seeding, *D:* without seeding) do not capture lower perplexities (grey boxes) at positions L52, L54 and L56 observed in PyIgClassify clusters.

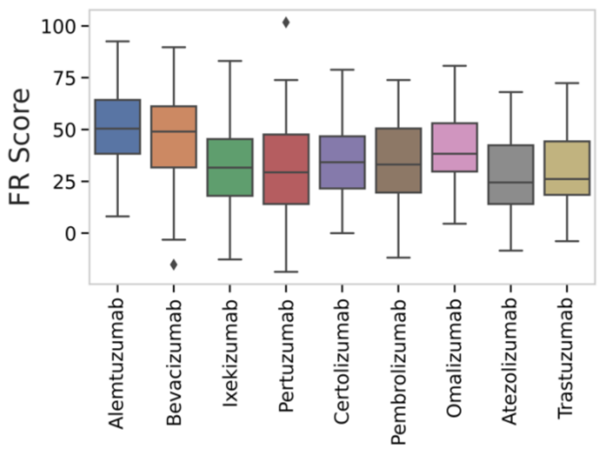

SI Figure 18. FR scores per design for hallucinated designs for the V_H-V_L interface for the humanized antibodies dataset.

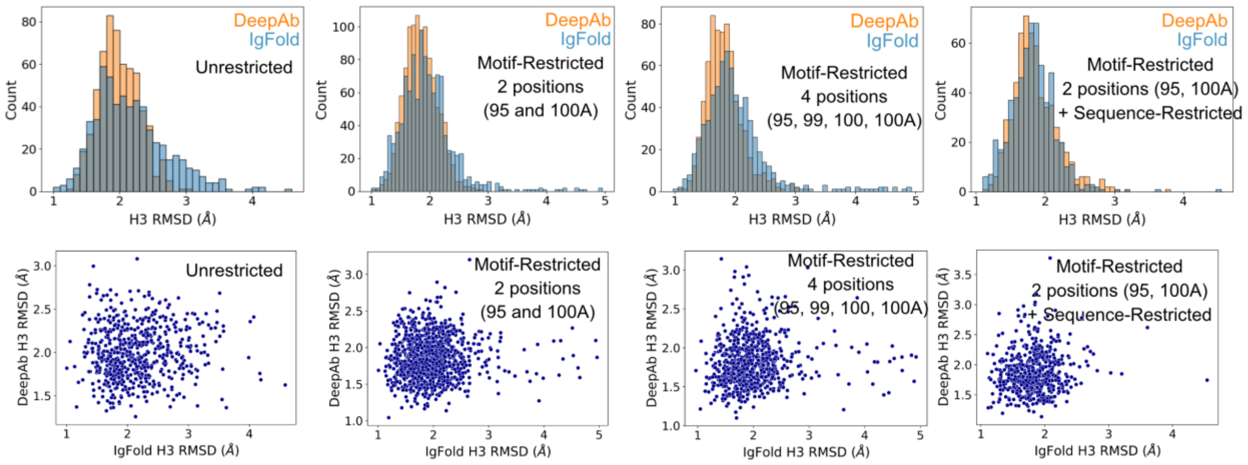

SI Figure 19. (Top) Distribution of H3 RMSD of the forward folded designs with DeepAb[1] and IgFold[2] from different hallucination runs for the Trastuzumab antibody. (Bottom) Comparison of IgFold and DeepAb RMSD's for hallucinated designs.

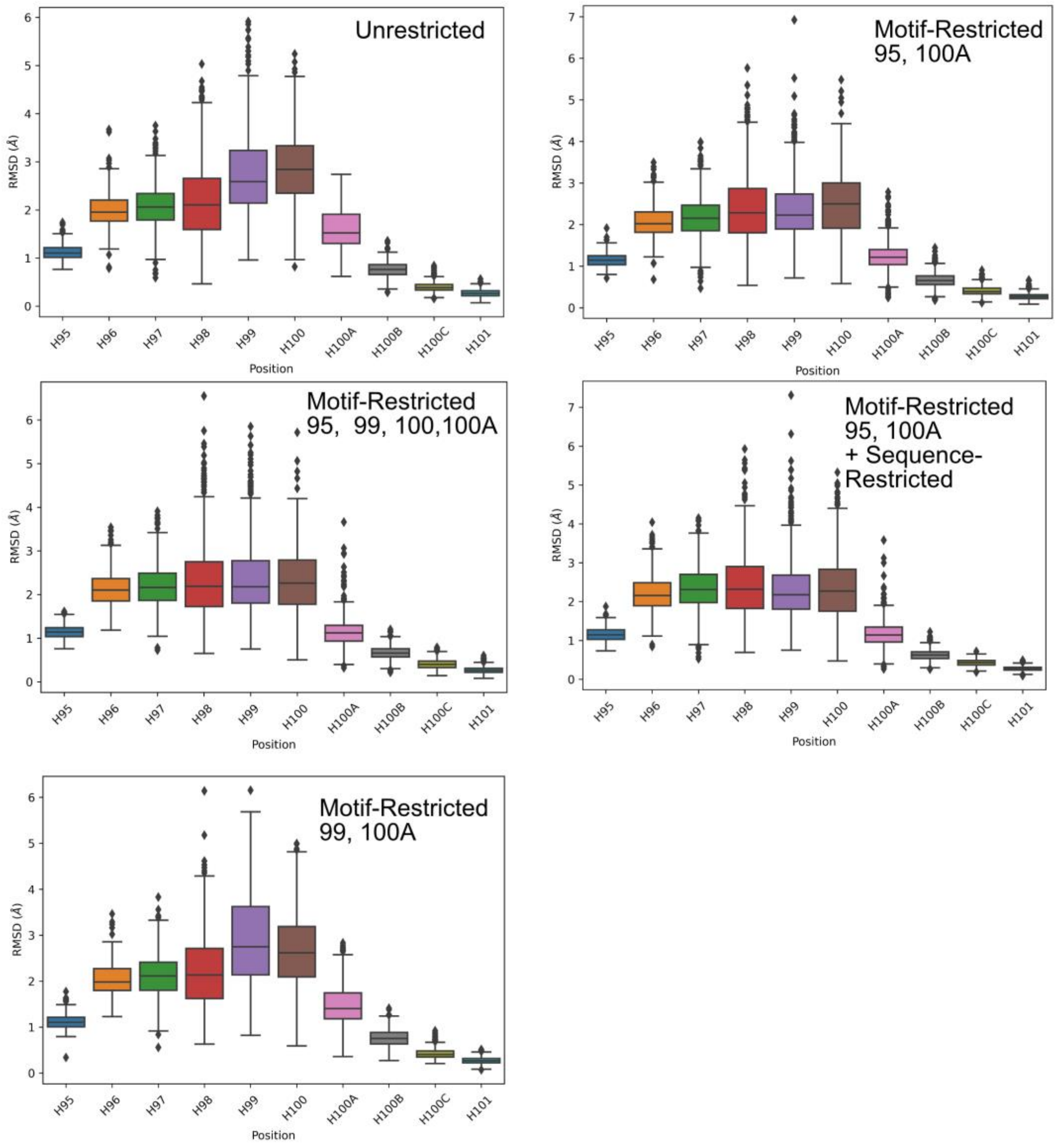

SI Figure 20. Distribution of per-residue backbone heavy atom RMSDs of forward folded designs from different hallucination runs for the Trastuzumab antibody.

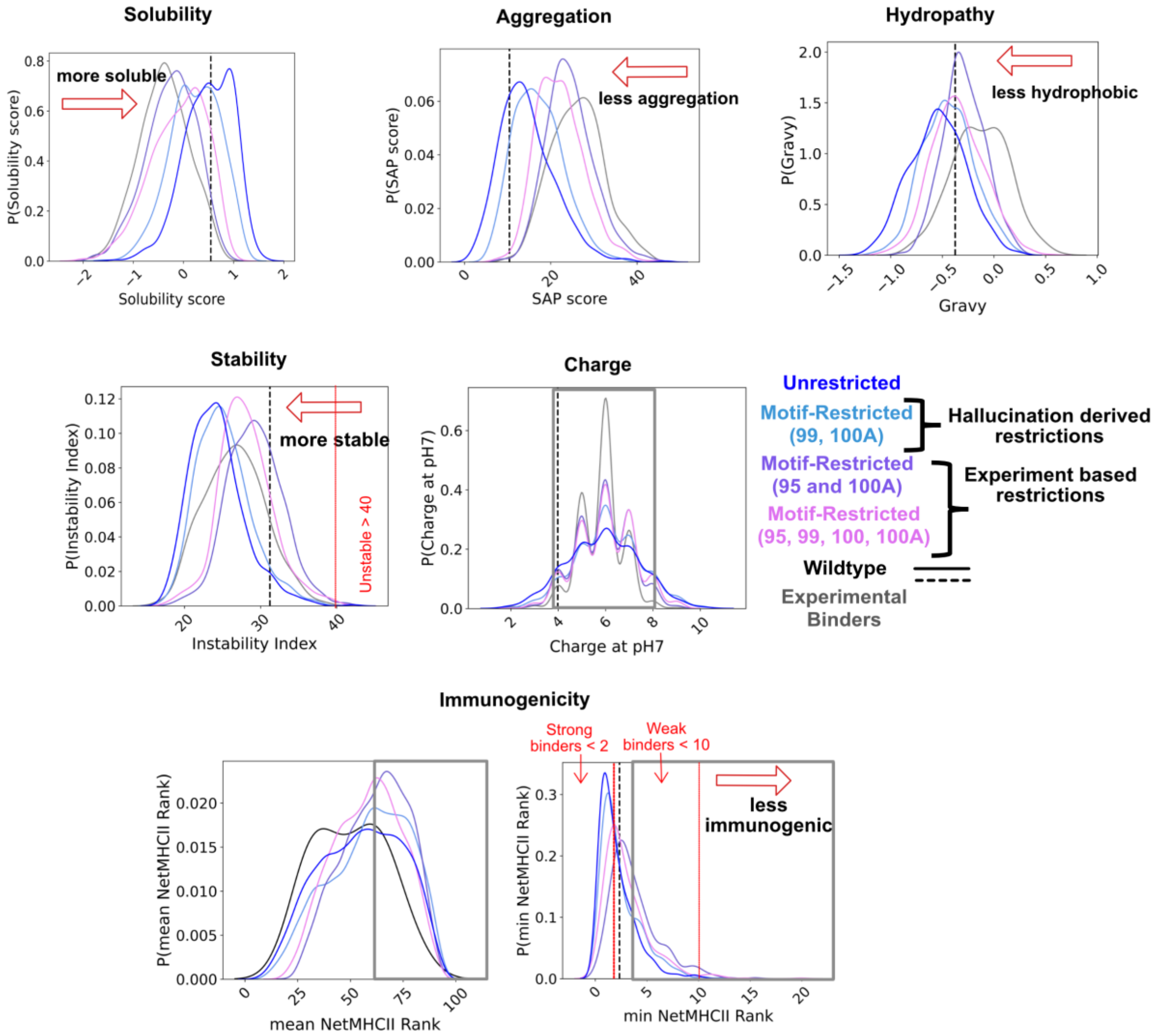

SI Figure 21. Distribution of developability metrics for hallucinated designs, 500 randomly selected experimental binders[3] and wildtype (*solid lines* for distributions and *dashed lines* for single sequence or structure). Min NetMHCII Rank refers to the lowest Rank for each design across all 26 alleles (see Methods) and across all 15-mer peptides (for that design) derived from the padded hallucinated sequences. Mean NetMHCII Rank refers to the average across all 26 alleles for all 15-mer peptides derived from the padded design sequences. For SAP score calculations, we used DeepAb folded structures. Grey boxes indicate windows of favorable metrics identified in Mason et al.[3]. For all plots except immunogenicity, we show metric scores for 500 randomly selected experimental binders. Red lines indicate NetMHCII Rank values for strong (< 2) and weak binders (< 10).

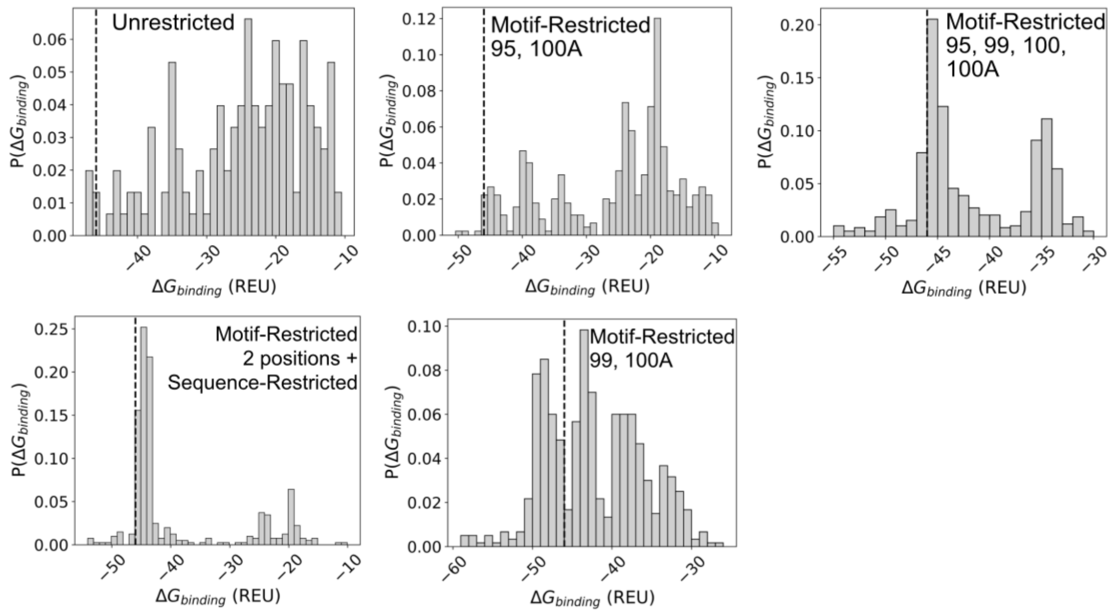

SI Figure 22. Distribution of $\Delta G_{binding}$ between HER2 and designs from different hallucination runs for the Trastuzumab antibody. Wildtype ΔG is indicated by the dashed line.

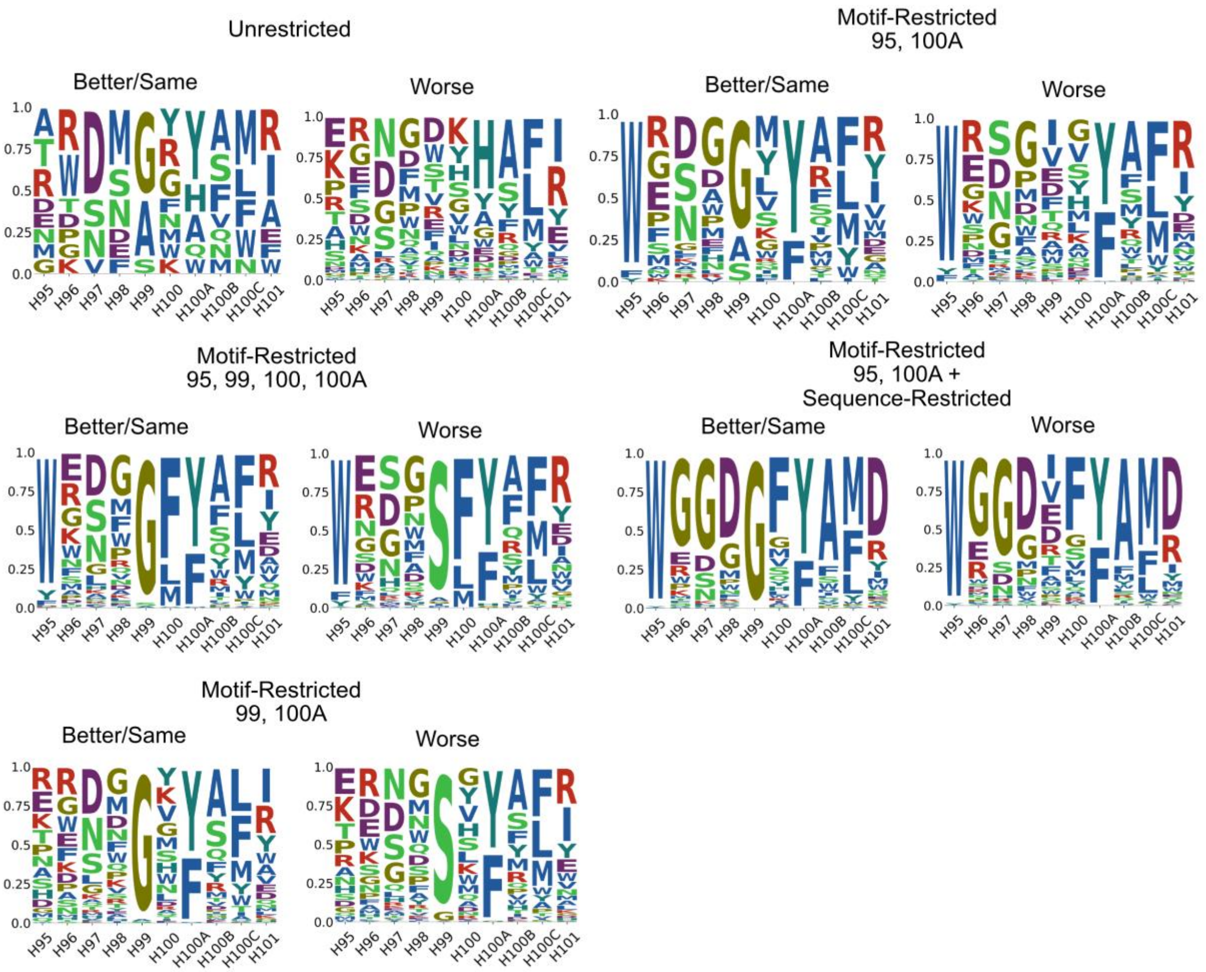

SI Figure 23. Sequence logos for designs with binding free energy either "Better/Same" as the wildtype or "Worse" than the wildtype after virtual screening for hallucinated libraries in different hallucination modes for the Trastuzumab antibody CDR H3.

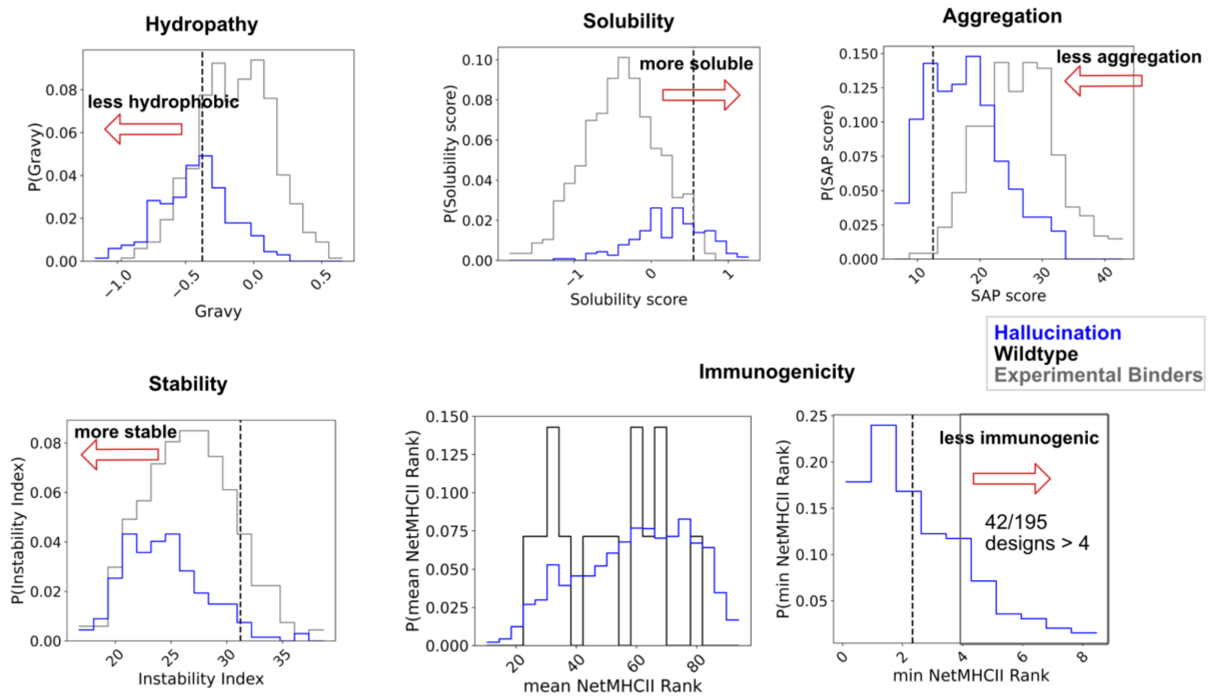

SI Figure 24. Distribution of various developability metrics for hallucinated and screened designs (with 99, 100A positions restricted), 500 randomly selected experimental binders[3] **and wildtype (*solid lines* for distributions and *dashed lines* for single sequence or structure). Grey boxes indicate windows of favorable metrics identified in Mason et al.**[3]**. For all plots except immunogenicity, we show metric scores for 500 randomly selected experimental binders.**

1. Ruffolo JA, Sulam J, Gray JJ. Antibody structure prediction using interpretable deep learning. Patterns. 2022;3: 100406. doi:10.1016/j.patter.2021.100406
2. Ruffolo JA, Chu L-S, Mahajan SP, Gray JJ. Fast, accurate antibody structure prediction from deep learning on massive set of natural antibodies. bioRxiv. 2022; 2022.04.20.488972. doi:10.1101/2022.04.20.488972
3. Mason DM, Friedensohn S, Weber CR, Jordi C, Wagner B, Meng SM, et al. Optimization of therapeutic antibodies by predicting antigen specificity from antibody sequence via deep learning. Nat Biomed Eng. 2021;5: 600–612. doi:10.1038/s41551-021-00699-9
